# The effects of cardiac structure, valvular regurgitation, and left ventricular diastolic dysfunction on the diagnostic accuracy of Murray law–based quantitative flow ratio

**DOI:** 10.3389/fcvm.2023.1134623

**Published:** 2023-05-24

**Authors:** Junqing Yang, Yuming Huang, Xiaoshan Li, Qianjun Jia, Huiliang Deng, Nianjin Xie, Meiping Huang, Hongwen Fei

**Affiliations:** ^1^ Department of Cardiology, Guangdong Provincial Key Laboratory of Coronary Heart Disease Prevention, Guangdong Cardiovascular Institute, Guangdong Provincial People's Hospital (Guangdong Academy of Medical Sciences), Southern Medical University, Guangzhou, China; ^2^ Department of Catheterization Lab, Guangdong Cardiovascular Institute, Guangdong Provincial Key Laboratory of South China Structural Heart Disease, Guangdong Provincial People’s Hospital (Guangdong Academy of Medical Sciences), Southern Medical University, Guangzhou, China; ^3^ Guangdong Medical University, Zhanjiang, China; ^4^ Guangdong Cardiovascular Institute, Guangdong Provincial People’s Hospital (Guangdong Academy of Medical Sciences), Southern Medical University, Guangzhou, China

**Keywords:** coronary hemodynamics, coronary heart disease, echocardiography, fractional flow reserve, quantitative flow ratio

## Abstract

**Objective:**

The study aimed to investigate the diagnostic accuracy of Murray law–based quantitative flow ratio (μQFR) from a single angiographic view in patients with abnormal cardiac structure, left ventricular diastolic dysfunction, and valvular regurgitation.

**Background:**

μQFR is a novel fluid dynamics method for deriving fractional flow reserve (FFR). In addition, current studies of μQFR mainly analyzed patients with normal cardiac structure and function. The accuracy of μQFR when patients had abnormal cardiac structure, left ventricular diastolic dysfunction, and valvular regurgitation has not been clear.

**Methods:**

This study retrospectively analyzed 261 patients with 286 vessels that underwent both FFR and μQFR prior to intervention. The cardiac structure and function were measured using echocardiography. Pressure wire–derived FFR ≤0.80 was defined as hemodynamically significant coronary stenosis.

**Results:**

μQFR had a moderate correlation with FFR (*r* = 0.73, *p* < 0.001), and the Bland–Altman plot presented no difference between the μQFR and FFR (0.006 ± 0.075, *p* = 0.192). With FFR as the standard, the diagnostic accuracy, sensitivity, specificity, positive predictive value, and negative predictive value of μQFR were 94.06% (90.65–96.50), 82.56% (72.87–89.90), 99.00% (96.44–99.88), 97.26 (89.91–99.30), and 92.96% (89.29–95.44), respectively. The concordance of μQFR/FFR was not associated with abnormal cardiac structure, valvular regurgitation (aortic valve, mitral valve, and tricuspid valve), and left ventricular diastolic function. Coronary hemodynamics showed no difference between normality and abnormality of cardiac structure and left ventricular diastolic function. Coronary hemodynamics demonstrated no difference among valvular regurgitation (none, mild, moderate, or severe).

**Conclusion:**

μQFR showed an excellent agreement with FFR. The effect of abnormal cardiac structure, valvular regurgitation, and left ventricular diastolic function did not correlate with the diagnostic accuracy of μQFR. Coronary hemodynamics showed no difference in patients with abnormal cardiac structure, valvular regurgitation, and left ventricular diastolic function.

## 1. Introduction

Fractional flow reserve (FFR) is the reference standard method for evaluating the physiological significance of non-occlusive coronary stenosis (1). FFR is defined as the ratio of distal pressure to aortic pressure determined during adenosine triphosphate–induced hyperemia. However, the clinical applications of FFR are limited due to the cost of pressure wire and the chest discomfort associated with hyperemia (1, 2).

Computational fluid dynamics (CFD) calculate the hemodynamics of the target organ or vessel with a medical image, avoiding invasive procedures (3). Quantitative flow ratio (QFR) is a novel method based on three-dimensional reconstruction and fluid dynamics algorithms from two angiographic views, which was extensively validated in coronary functional evaluation. The Murray law–based quantitative flow ratio (μQFR) is a novel computational approach based on Murray’s law, wherein bifurcation lesions caused the target vessel’s pressure to decrease. A single angiographic view–based μQFR was reported with technical advantages, such as simpler operation, shorter analysis time, and better reproducibility (4–6). However, the blood becomes flow eddies in an abnormal cardiac structure, especially the enlargement of the atrium and ventricle, which disturbs cardiac hemodynamics. When the left ventricle becomes enlarged, abnormal cardiac hemodynamics lead to abnormal coronary hemodynamics. Valvular regurgitation affects cardiac hemodynamics by the return of blood, which flows back into the atrium or ventricle. Aortic regurgitation changes the hemodynamics of the aorta, which influence coronary hemodynamics. The left ventricular diastolic dysfunction means that the left ventricle cannot discharge blood normally, affecting the hemodynamics of the aorta and coronary. Some studies had been reported that the hemodynamics of coronary in patients with aortic valve stenosis have changed (7, 8). The diagnostic accuracy of μQFR with abnormal cardiac structure, valve regurgitation, or left ventricular diastolic dysfunction has not been reported previously. The present study aimed to study the effects of abnormal cardiac structure, valvular regurgitation, and left ventricular diastolic dysfunction on the diagnostic accuracy of μQFR.

## 2. Materials and methods

### 2.1. Study population

**Figure 1:**
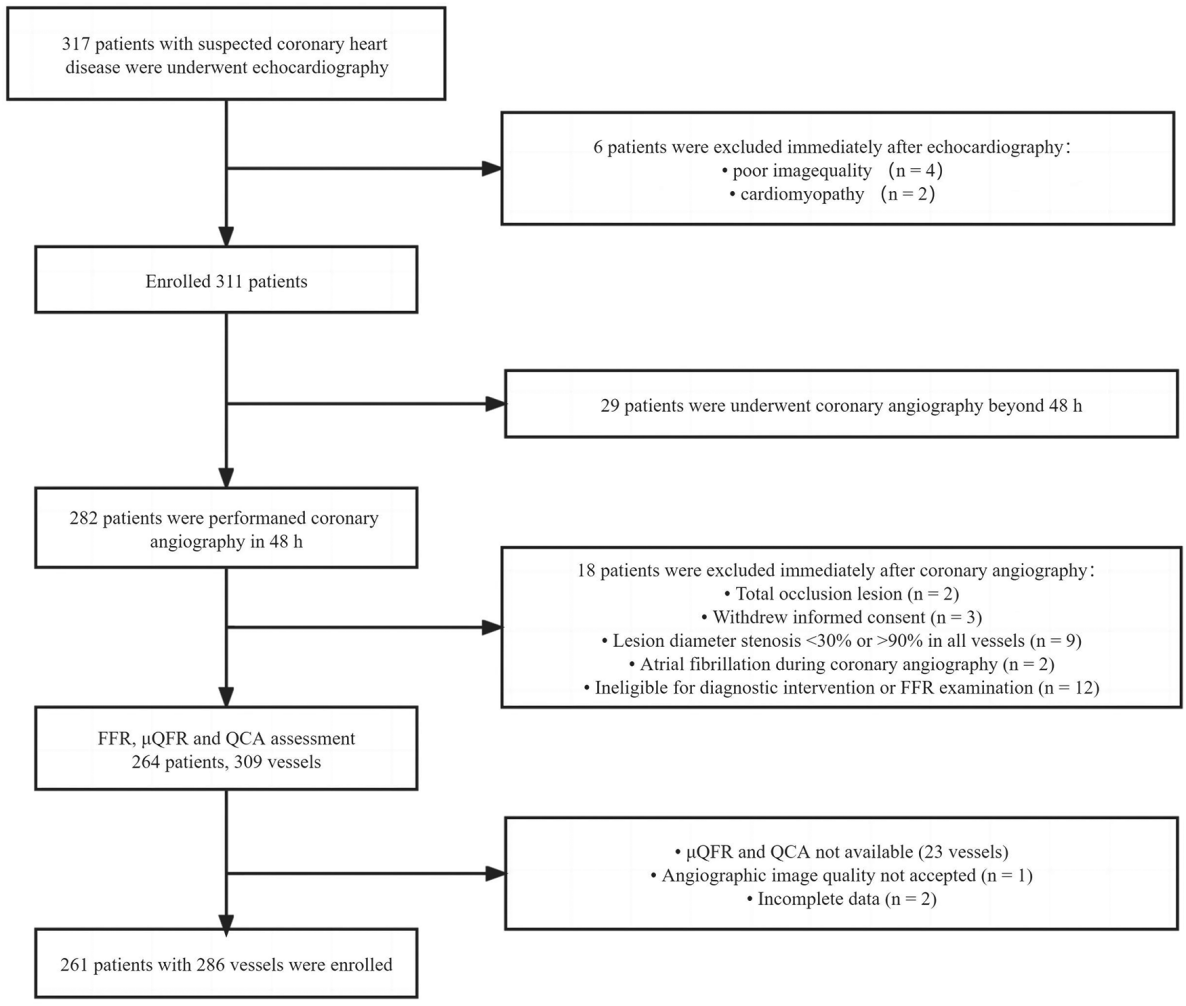
Study flowchart. QCA, quantitative coronary angiography; FFR, fractional flow reserve; μQFR, Murray law–based quantitative flow ratio.

This study retrospectively analyzed patients who underwent coronary angiography and echocardiography for suspected coronary heart disease in Guangdong Provincial People's Hospital between March 2016 and November 2018. Patients who underwent FFR measurement for the evaluation of myocardial ischemia were enrolled. Target vessels (≥2 mm) with percentage diameter stenosis (DS) between 30% and 90% were found in quantitative coronary angiography (QCA), and patients who underwent echocardiography, which was performed within 2 days before coronary angiography, were included. The left main trunk, bypass graft lesions, a poor-quality coronary angiogram for μQFR and QCA (e.g., foreshortening or overlap of the culprit vessels, insufficient contrast flush, frequent atrial premature, or atrial fibrillation), cardiomyopathy, and patients whose echocardiography could not be demonstrated due to poor acoustic window were excluded. All the patients in this study were exempted from writing informed consent, and the Research Ethics Committee of Guangdong Provincial People's Hospital approved this study. The study flowchart is shown in Figure 1.

### 2.2. Coronary angiography and QCA analysis

A digital subtraction angiography machine (Allura, Philips, Amsterdam, Netherlands) performed coronary angiography, and angiography images were recorded at 15 frames/s. The field of view (FOV) was 20 cm × 20 cm − 22 cm × 22 cm, the matrix was 512 × 512, the tube current was 500−800 mA, and the tube voltage was 60−120 kV. The non-ionic contrast agent with an iodine content of 350−370 mgI/ml was injected. Before coronary angiography, nitroglycerin was injected into the target vessel to exclude coronary spasms caused by medical devices. An experienced technician using QCA software (Beijing Crealife Technology Co., Ltd., Beijing, China) selected the angiogram in the diastolic period. Meanwhile, the technician analyzed the quantitative coronary angiography values of the target vessels. QCA software automatically delineated the lumen contour of the target vessels, and a manual correction was allowed when the error of measurement existed. An intermediate QCA technician was selected to analyze the QCA; after that, a senior technician verified all data.

### 2.3. FFR analysis

Pressure wire–based FFR was determined by a RadiAnalyzer Xpress instrument and Certus pressure wire (St. Jude Medical, St. Paul, MN, United States). Adenosine-5′-triphosphate (ATP) was injected to induce hyperemia via the elbow vein at ≥60 μg/L/min. The pressure sensor was drawn back to the catheter tip to exclude the pressure drift of the Certus pressure wire; a pressure drift between −0.03 and 0.03 was accepted during hyperemia. Otherwise, the FFR performance had to be redone.

### 2.4. μQFR analysis

A μQFR analysis was performed by the Pulse Medical software (Pulse Medical Imaging Technology Shanghai, Shanghai, China). A well-trained technician selected the diastolic period for μQFR analysis. Delineation of the lumen contour of the target vessels was carried out by the software, and a manual correction was allowed when the identified lumen contour was wrong. Before μQFR analysis, the stenosis segments of vessels were marked up to ensure that the μQFR and QCA could be compared with FFR at the same site.

### 2.5. Echocardiographic parameters

All transthoracic 2D echocardiograms were performed by experienced operators using ultrasound machines with an ultrasonic probe frequency between 2.5 and 5.5 MHz (Vivid series, GE Healthcare or EPIQ series, Philips Medical). All echocardiographic parameters were recorded according to the American Society of Echocardiography (ASE) (9).

Patients were in the left lateral position. The linear internal measurements of the left ventricle, interventricular septum, and posterior wall were measured in the parasternal long-axis view. The interventricular septum at end diastole (IVSd), left ventricular end-diastolic diameter (LVEDD), left ventricular posterior wall at end diastole (LVPWd), and left ventricular end-systolic diameter (LVESD) were obtained perpendicular to the left ventricular long axis and measured below the level of the mitral valve leaflet tips. The anteroposterior diameter of the left atrium was measured in the parasternal long-axis view perpendicular to the aortic root long axis and measured at the level of the aortic sinuses. The right atrial and right ventricular diameters were measured in the apical 4 chamber view. Left ventricular ejection fraction (LVEF) was estimated by the biplane Simpson method in the apical four-chamber and apical two-chamber views. The cardiac structure was considered abnormal if one or more of the following criteria were met: (1) left ventricular end-diastolic diameter ≥54 mm; (2) left atrial internal diameter ≥38 mm; (3) right atrial length diameter ≥53 mm; and (4) right ventricular length diameter ≥75 mm (9).

The diastolic function of the left ventricle was assessed by a two-dimensional image of the left ventricle and tissue Doppler imaging of the septal mitral annulus and transmittal blood flow velocities (10).

The valvular regurgitation severity was evaluated by color flow Doppler according to the regurgitant jet area (11). The condition of mitral and tricuspid regurgitation was graded as “none” in the absence of any detectable regurgitant jet or jet area less than 1.5 cm^2^, whereas a jet area less than 4 cm^2^ was graded as “mild” (12). A jet area between 4 and 8 cm^2^ was graded as “moderate,” while a jet area more than 8 cm^2^ was graded as “severe.” The condition of aortic regurgitation was graded as “none” in the absence of any detectable regurgitant jet or jet area less than 1.0 cm^2^, whereas a jet area less than 4 cm^2^ was graded as “mild,” a jet area between 4 and 8 cm^2^ as “moderate,” and a jet area more than 8 cm^2^ as “severe” (13–15).

### 2.6. Statistical analysis

Continuous variables were expressed as mean ± standard deviation and compared with *t*-tests for normally distributed variables by *post hoc* analysis or Mann–Whitney *U*-tests for non-normally distributed variables by *post hoc* analysis and one-way analysis of variance. Categorical variables were expressed as frequencies and compared with *χ*^2^ and Fisher's exact tests. Our study used the FFR as the gold standard, and the diagnostic accuracy of μQFR was determined by calculating the sensitivity, specificity, positive predictive value (PPV), negative predictive value (NPV), positive likelihood ratio (+LR), and negative likelihood ratio (−LR), as appropriate. Meanwhile, we calculated the diagnostic accuracy of μQFR in abnormal and normal cardiac structures. The accuracy of μQFR was provided with two-sided 95% confidence intervals (CIs). When the FFR and μQFR values were less than or equal to 0.80, the functional evaluation of coronary stenosis was significant, whereas when the FFR and μQFR values were more than 0.80, the functional evaluation of coronary stenosis was non-significant. The correlation between FFR and μQFR was determined by Spearman's correlation coefficient (*r*). The difference between μQFR and FFR was reported using a Bland–Altman plot. Our team used the μQFR/FFR concordance to define the vessel with the same coronary hemodynamics. Using the FFR as the reference standard, we divided μQFR/FFR into two groups according to μQFR/FFR concordance and disconcordance. The receiver operating curve (ROC) was used to calculate the area under the curve of μQFR. All the statistical analyses were performed with MedCalc (version 14.12.0, MedCalc Software, Ostend, Belgium). A two-sided *p* < 0.05 was considered statistically significant.

## 3. Results

### 3.1. Baseline clinical and lesion characteristics

From March 2016 to November 2018, a total of 261 patients with 286 interrogated vessels were enrolled, with a mean age of 62.25 ± 9.40 years. A total of 190 (72.80%) patients were male, 74 (28.35%) patients had diabetes mellitus, and 150 (57.47%) patients had hypertension. There were 31 (11.88%) patients with hyperlipidemia, 72 (27.59%) patients were smoking, six patients had a family history of coronary heart disease, four patients had a previous myocardial infarction, 52 patients had previous percutaneous coronary intervention (PCI) history, two patients had coronary artery bypass grafting (CABG) history (LVEF was 63.65 ± 8.88%), and 216 patients had left ventricular diastolic dysfunction. The basic clinical characteristics of the selected patients are shown in Table 1.

**Table 1:** Baseline clinical characteristics.

	*n* = 261
Age, years	62.25 ± 9.40
Male	190 (72.80%)
Diabetes mellitus	74 (28.35%)
Hypertension	150 (57.47%)
Hyperlipidemia	31 (11.88%)
Current smoker	72 (27.59%)
Family history of coronary artery disease	6 (2.30%)
Previous myocardial infarction	4 (1.53%)
History of percutaneous coronary intervention	52 (19.92%)
History of coronary artery bypass grafting	2 (0.77%)
Diameter of the left atrium, mm	35.18 ± 4.91
Left ventricular end-diastolic diameter, mm	46.80 ± 5.56
Diameter of the right atrium, mm	45.87 ± 24.76
Diameter of right ventricular, mm	50.25 ± 4.74
Left ventricular ejection fraction, %	63.65 ± 8.88
Left ventricular diastolic dysfunction	216 (82.76%)

Continuous values are mean ± SD. Categorical values are *n* (%).

**Figure 2:**
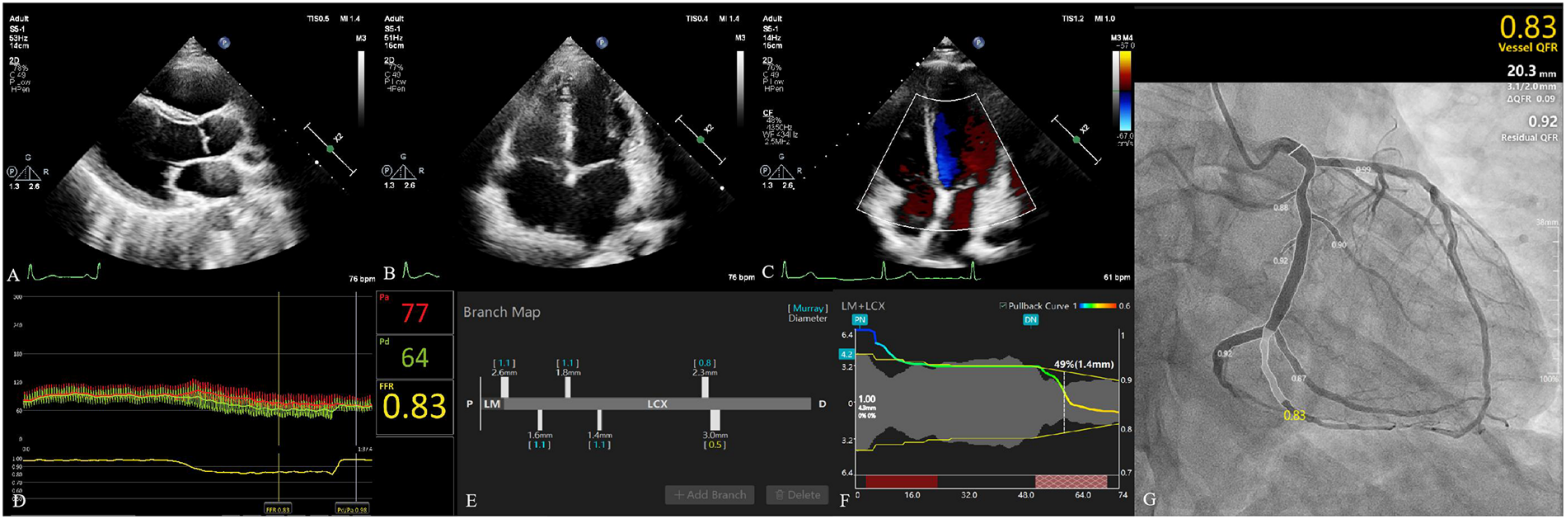
Evaluation of cardiac structure, valvular regurgitation, left ventricular diastolic dysfunction, and coronary hemodynamic. (**A**) The parasternal left ventricular long-axis view. The left atrium diameter was 28 mm. Left ventricular end-diastolic diameter and left ventricular end-systolic diameter were 44 and 24 mm, respectively. (**B**) The apical four-chamber view. The diameter of the right atrium and right ventricle was 40 and 49 mm, respectively. (**C**) Evaluation of valvular regurgitation by transthoracic Doppler echocardiography. (**D**) The fractional flow reserve of LCX was 0.83. (**E**) The branch map of LCX. (**F**) The pullback curve of LCX; the diameter stenosis in μQFR was 49%. (**G**) The μQFR of LCX was 0.83. LCX, left circumflex artery; μQFR, Murray law–based quantitative flow ratio.

Vessel characteristics are presented in Table 2. The most common vessel was the left anterior descending artery (LAD) (194, 67.83%). FFR and μQFR had mean values of 0.83 ± 0.08 and 0.84 ± 0.11, respectively. FFR ≤0.80 was noted in 86 (30.07%) vessels, while μQFR ≤0.80 was noted in 73 (25.52%) vessels. The DS in QCA had a mean value of 47.31 ± 12.21%. In addition, 16.78% of vessels were bifurcation lesions, 10.49% of vessels were tortuous lesions, and 2.80% of vessels were moderate or severe calcified lesions. The evaluation of cardiac structure, valvular regurgitation, left ventricular diastolic dysfunction and coronary hemodynamic is shown in Figure 2.

**Table 2:** Vessel characteristics.

	*n* = 286
Left anterior descending artery	194 (67.83%)
Left circumflex artery	40 (13.99%)
Right coronary artery	49 (17.13%)
Obtuse marginal branch	1 (0.35%)
Diagonal branch	2 (0.70%)
Reference vessel diameter, mm	3.19 ± 0.70
Diameter stenosis in QCA, %	47.31 ± 12.21
Bifurcation lesions	48 (16.78%)
Tortuous lesions	30 (10.49%)
Moderate or severe calcified lesions	8 (2.80%)
Thrombotic lesions	1 (0.35%)
Tandem lesions	41 (14.34%)
FFR (per vessel)	0.83 ± 0.08
Vessels with FFR ≤0.80	86 (30.07%)
μQFR (per vessel)	0.84 ± 0.11
Vessels with μQFR ≤0.80	73 (25.52%)

QCA, quantitative coronary angiography; FFR, fractional flow reserve; μQFR, Murray law–based quantitative flow ratio.

Continuous values are mean ± SD. Categorical values are *n* (%).

### 3.2. Correction and agreement between FFR and μQFR

**Figure 3:**
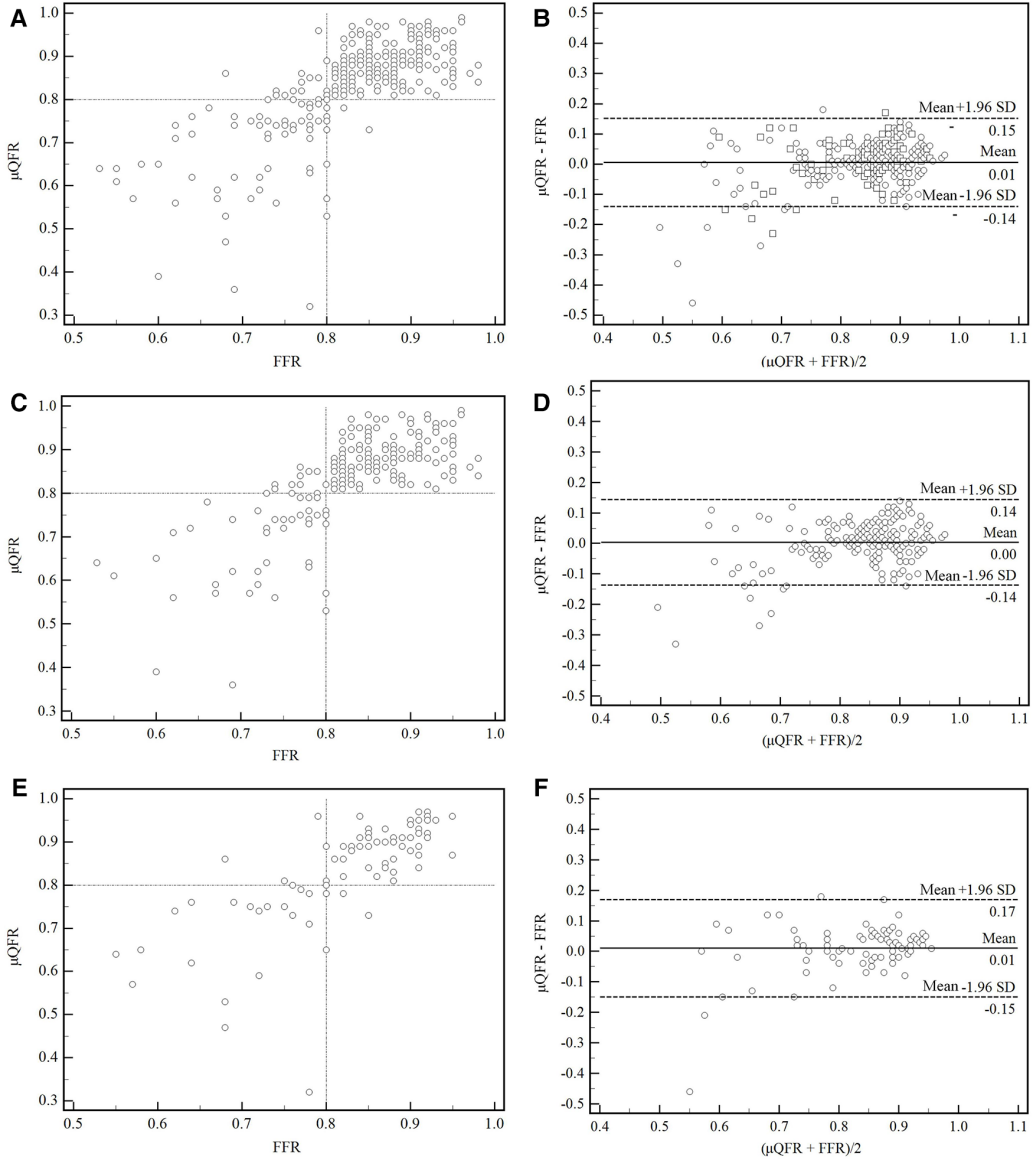
Correction and agreement between FFR and μQFR. (**A**) Linear regression between FFR and μQFR in all vessels. (**B**) The Bland–Altman plot presented a difference between the FFR and μQFR in all vessels. (**C,D**) Linear regression and Bland–Altman plot presented a difference between FFR and μQFR in normal cardiac structure. (**E,F**) Linear regression and Bland–Altman plot presented a difference between FFR and μQFR in abnormal cardiac structure. SD, standard deviation; FFR, fractional flow reserve; μQFR, Murray law–based quantitative flow ratio.

A moderate correlation between FFR and μQFR was found in all vessels, in normal and abnormal cardiac structures (*r* = 0.73, 95% CI: 0.67–0.78, *p* < 0.001; *r* = 0.71, 95% CI: 0.63–0.77, *p* < 0.001; *r* = 0.77, 95% CI: 0.67–0.84, *p* < 0.001). No significant difference was found between FFR and μQFR in all vessels, in normal and abnormal cardiac structures, as shown by the Bland–Altman analysis (0.006 ± 0.075, *p* = 0.192; 0.004 ± 0.069, *p* = 0.458; 0.010 ± 0.0815, *p* = 0.241) (Figure 3).

**Figure 4:**
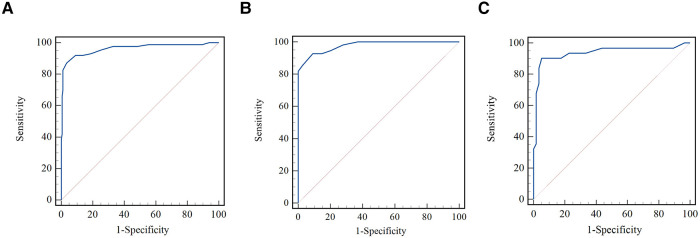
Comparison of receiver operating curves for the discrimination of coronary hemodynamic deficiency. (**A**) Comparison of receiver operating curves for all vessels. (**B**) Comparison of receiver operating curves for normal cardiac structure. (**C**) Comparison of receiver operating curves for abnormal cardiac structure.

As shown in Figure 4, the μQFR showed an excellent predictive value for coronary hemodynamic deficiency in all vessels, in normal and abnormal cardiac structures [all, area under the receiver operating characteristic curve (AUC) = 0.96; normal cardiac structure, AUC = 0.94; abnormal cardiac structure, AUC = 0.98].

With an FFR cutoff value (≤0.80) to define hemodynamically significant lesions, the per-vessel diagnostic accuracy of μQFR was 94.06 (95% CI: 90.65–96.50), with 71 true positives, 198 true negatives, 2 false positives, and 15 false negatives. Sensitivity, specificity, PPV, NPV, (+) LR, and (−) LR of μQFR were 82.56% (95% CI: 72.87–89.90), 99.00% (95% CI: 96.44–99.88), 97.26 (95% CI: 89.91–99.30), 92.96% (95% CI: 89.29–95.44), 82.56 (95% CI: 20.72–328.95), and 0.18 (95% CI: 0.11–0.28), respectively. The diagnostic accuracy of μQFR in normal and abnormal cardiac structures is shown in Table 3.

**Table 3:** Diagnostic performance of μQFR.

	All (95% CI)	Abnormal cardiac structure (95% *CI*)	Normal cardiac structure (95% CI)
Accuracy, %	94.06 (90.65–96.50)	92.05 (84.30–96.74)	94.95 (90.91–97.55)
Sensitivity, %	82.56 (72.87–89.90)	83.87 (66.27–94.55)	81.82 (69.10–90.92)
Specificity, %	99.00 (96.44–99.88)	96.49 (87.89–99.57)	100 (97.45–100.00)
PPV, %	97.26 (89.91–99.30)	92.86 (76.76–98.08)	100.00
NPV, %	92.96 (89.29–95.44)	91.67 (83.11–96.09)	93.46 (89.09–96.16)
(+) LR	82.56 (20.72–328.95)	23.90 (6.07–94.08)	–
(−) LR	0.18 (0.11–0.28)	0.17 (0.08–0.37)	0.18 (0.10–0.32)

CI, confidence interval; (+) LR, positive likelihood ratio; (−) LR, negative likelihood ratio; NPV, negative predictive value; PPV, positive predictive value; AUC, area under the receiver operating characteristic curve.

Values are *n* (95% CI) for (+) LR, (−) LR, and AUC and *n*% (95% CI) for all other parameters.

### 3.3. Impact of vessels, cardiac structure, valvular regurgitation, and left ventricular diastolic function on concordance between μQFR and FFR

The hemodynamic concordance was not associated with vessels, left ventricular diastolic function, and abnormal cardiac structure (*p* > 0.05). In terms of valvular regurgitation (aortic, mitral, and tricuspid valve) grade, there was no difference between the two groups (*p* > 0.05). The diagnostic accuracy of μQFR was not associated with vessels, left ventricular diastolic function, abnormal cardiac structure, and valvular regurgitation (Table 4).

**Table 4:** Vessel, cardiac structure, and valvular regurgitation characteristics with μQFR/FFR concordance or disconcordance.

μQFR/FFR concordance	Concordance	Disconcordance	*p*
*n*	269	17	
Vessel			0.347
Left anterior descending artery	179 (66.54%)	15 (88.24%)	
Left circumflex artery	38 (14.13%)	2 (11.76%)	
Right coronary artery	49 (18.22%)	0 (0.00%)	
Diagonal branch	2 (0.74%)	0 (0.00%)	
Obtuse marginal branch	1 (0.37%)	0 (0.00%)	
Left atrium diameter, mm	35.04 ± 4.98	36.00 ± 4.72	0.439
Left ventricular end-systolic diameter, mm	29.91 ± 5.76	32.53 ± 9.06	0.081
Left ventricular end-diastolic dimension, mm	46.83 ± 5.38	48.35 ± 7.57	0.272
Right atrium diameter, mm	45.70 ± 24.45	45.35 ± 6.42	0.953
Right ventricular diameter, mm	50.09 ± 4.77	50.76 ± 3.25	0.565
Interventricular septum, mm	10.25 ± 1.57	10.49 ± 1.07	0.535
Left ventricular posterior wall, mm	9.96 ± 1.34	10.29 ± 0.90	0.308
E/e′	12.2 ± 4.6	12.5 ± 3.2	0.445
Left ventricular ejection fraction, %	63.74 ± 8.65	61.65 ± 10.98	0.342
Cardiac structure			0.339
Normal	188 (69.89%)	10 (58.82%)	
Abnormal	81 (30.11%)	7 (41.18%)	
Left ventricular diastolic function			0.890
Normal	44 (16.36%)	3 (17.65%)	
Abnormal	225 (83.64%)	14 (82.35%)	
Aortic regurgitation grade			0.365
None	192 (71.38%)	10 (58.82%)	
Mild	72 (26.77%)	6 (35.29%)	
Moderate or severe	5 (1.86%)	1 (5.88%)	
Mitral regurgitation grade			0.616
None	182 (67.66%)	10 (58.82%)	
Mild	70 (26.02%)	5 (29.41%)	
Moderate or severe	17 (6.32%)	2 (11.76%)	
Tricuspid regurgitation grade			0.442
None	195 (72.49%)	11 (64.71%)	
Mild	64 (23.79%)	6 (35.29%)	
Moderate or severe	10 (3.72%)	0 (0.00%)	

E, mitral flow early velocity; e′, septal early diastolic mitral annulus velocity; E/e′, mitral inflow to mitral relaxation velocity ratio; FFR, fractional flow reserve; μQFR, Murray law–based quantitative flow ratio.

Continuous values are mean ± SD. Categorical values are *n* (%).

### 3.4. Impact of cardiac structure, valvular regurgitation, and left ventricular diastolic function on μQFR and FFR

No statistically significant difference was found in FFR and μQFR with an abnormal cardiac structure and left ventricular diastolic function (*p* > 0.05). Likewise, FFR and μQFR indicated that the difference was not statistically significant in valvular regurgitation (aortic, mitral, and tricuspid valve) (*p* > 0.05) (Table 5).

**Table 5:** Coronary hemodynamics with cardiac structure and valvular regurgitation characteristics.

	None	Mild	Moderate or severe	*p*
Aortic regurgitation				
*n*	202	78	6	
FFR	0.83 ± 0.08	0.83 ± 0.08	0.74 ± 0.14	0.206
μQFR	0.84 ± 0.11	0.84 ± 0.11	0.74 ± 0.17	0.121
Mitral regurgitation				
*n*	192	75	19	
FFR	0.83 ± 0.08	0.83 ± 0.09	0.82 ± 0.11	0.949
μQFR	0.84 ± 0.10	0.83 ± 0.13	0.83 ± 0.13	0.696
Tricuspid regurgitation				
*n*	206	70	10	
FFR	0.83 ± 0.09	0.83 ± 0.08	0.86 ± 0.06	0.465
μQFR	0.84 ± 0.11	0.83 ± 0.10	0.85 ± 0.12	0.950
	Normal	Abnormal		
Cardiac structure				
*n*	198	88		
FFR	0.83 ± 0.08	0.82 ± 0.09		0.318
μQFR	0.84 ± 0.11	0.83 ± 0.12		0.755
Left ventricular diastolic function				
*n*	47	239		
FFR	0.83 ± 0.08	0.83 ± 0.09		0.884
μQFR	0.83 ± 0.13	0.84 ± 0.11		0.645

FFR, fractional flow reserve; μQFR, Murray law–based quantitative flow ratio.

Continuous values are mean ± SD. Categorical values are *n* (%).

## 4. Discussion

In this research, we found the following: (1) With FFR as the gold standard, μQFR presented relatively precise accuracy with FFR (94.51%). Meanwhile, Bland–Altman analysis demonstrated no difference between μQFR and FFR (0.006 ± 0.077, *p* = 0.192). (2) The value of FFR had no difference in vessels, abnormal cardiac structure, valvular regurgitation, and left ventricular diastolic dysfunction. (3) The effects of abnormal cardiac structure, valvular regurgitation, and left ventricular diastolic dysfunction on the diagnostic accuracy of μQFR were low.

### 4.1. QFR with coronary hemodynamic insufficiency

Many studies had confirmed that the accuracy of QFR in identifying hemodynamically significant coronary stenosis was high, the AUC of QFR was between 0.93 and 0.97 (16). Tu et al. (4, 5) found out that the three-dimensional quantitative flow ratio (3D-QFR) and μQFR in identifying hemodynamic significant coronary stenosis were as high as 92.7% and 93.0%, which were similar to our study (94.5%). The correlation of the FAVOR Pilot study, WIFI II study, FAVOR II China study, and FAVOR II Europe–Japan study (17) was more than 0.70, which was similar to our study. The analysis time of μQFR was short (67 ± 22 s) (4), and the reproducibility was high. μQFR could determine the physiologically significant stenosis in the catheter laboratory. It's a theoretical possibility that, when the lesions were eccentric, a three-dimensional angiography reconstruction based on two angiography views was more accurate in quantifying the lumen area, avoiding the second view that presented that the vessel was foreshortening or overlapped (4).

### 4.2. QFR with vessel characteristics

A QFR showed a high diagnostic accuracy and correlation in the left anterior descending artery, left circumflex artery (LCX), and right coronary artery (RCA) (6). Meanwhile, QFR showed a low diagnostic accuracy and correlation in calcified lesions, tortuous lesions, bifurcated lesions, and coronary ostia. FFR was the gold standard for the assessment of borderline lesions, and the diagnostic accuracy and AUC of QFR were 85% and 0.93 (18). In FAVOR II China (6), FAVOR II Europe–Japan (19), and WIFI II (20), LAD was the most common vessel (56%, 51%, and 58%, respectively), and 33%–36% vessels had an FFR ≤0.80. The diagnostic accuracy of QFR was more than 80% in our study than that of the previous study (the percentage of LAD and the diagnostic accuracy were 68% and 95%, respectively). With FFR as the gold standard, there was no statistical difference between LAD, LCX, and RCA in the diagnostic accuracy of QFR (*p* = 0.347).

### 4.3. QFR with cardiac structure characteristics and left ventricular diastolic function

Most research in QFR focused on left ventricular ejection fraction without the information of cardiac structure in echocardiography. We found that there was no difference in QFR agreement with FFR (AUC: 0.91), when the cardiac structure was abnormal (*p* = 0.338).

Typically, the hemodynamics of vessels had a positive correlation with LVEF, the value of hemodynamics increased with the enhancement of LVEF, especially for vessels with FFR <0.80 (21). Zhong et al. (22) pointed out that QFR with LVEF less than 50% was lower than that of LVEF with more than 50%. In this paper, 83.57% (239) of patients with left ventricular diastolic dysfunction, while the mean value of LVEF was 63.65 ± 8.88%, and the accuracy of μQFR was as high as 94.1%. There was no difference between μQFR/FFR concordance and disconcordance in patients with left ventricular diastolic dysfunction. At the same time, there was no difference between left ventricular diastolic dysfunction and abnormal FFR and QFR.

### 4.4. QFR with valvular regurgitation

The mitral regurgitation generally does not influence the aortic pressure, especially the pressure of the coronary ostium, so there was no obvious evident impact of functionally significant coronary stenosis in mitral regurgitation. Scarsini et al. (23) reported a case of a patient with tricuspid regurgitation. When the patient underwent tricuspid valve replacement via catheter, an FFR was performed to evaluate functionally significant stenosis in coronary artery disease. It was found that the significant decrease in right atrial pressure did not affect the value of FFR. In our study, the diagnostic accuracy of μQFR was not related to mitral and tricuspid regurgitation (*p* = 0.621, *p* = 0.446). In the diagnosis of myocardial ischemia caused by the coronary artery, despite the mitral or tricuspid being regurgitant, μQFR showed high confirmed diagnostic consistency with FFR. Ge et al. (24) pointed out that coronary flow reserve (CFR) was more susceptible than FFR to the influence of aortic regurgitation and coronary artery disease. Coronary slow flow is considered one of the possible mechanisms of myocardial ischemia, which changes the microvascular perfusion of the coronary. This microvascular disease changes the coronary circulation, which aggravates the mismatch between the blood supply and demand, and then reducing the coronary flow reserve and causing myocardial ischemia and hypoxia. However, the correlation between the reduction of coronary perfusion pressure caused by aortic regurgitation and the coronary slow flow was unclear, and the change in coronary hemodynamics caused by aortic regurgitation has not been uncertain. In our study, no statistical difference was found in the accuracy of QFR between aortic valve normal and regurgitated (*p* = 0.370). In the diagnosis of myocardial ischemia caused by the coronary artery, μQFR showed high consistency with FFR.

### 4.5. Limitations of this study

First, this study was a single-center study; the sample size of aortic regurgitation, small vessel (the reference vessel diameter was less than 3 mm), and vessels with FFR ≤0.80 was small, and most of the vessels were LAD. Second, the sample size of the abnormal cardiac structure was moderate; most of the abnormalities were left atrium (75, 26.22%). Third, the prevalence of functional ischemia (30.07%) and abnormal structure were relatively low. Fourth, the QFR we used was a μQFR without 3D modeling, meaning that the accuracy of the vessel with eccentric plaque is low. Fifth, there was little information to discuss the application of QFR in patients with left ventricular diastolic dysfunction, and the practical further results need to be verified in future clinical tasks. Sixth, coronary hemodynamics evaluation is mainly applied to patients with aortic stenosis, and little information study patients with aortic regurgitation. At the same time, the sample size of aortic regurgitation in our study is small, and the practical further results need to be verified in future clinical tasks. Seventh, we did not calculate QFR from the second view; we only used one view that best exposed the contour of the vessels to calculate QFR.

## 5. Conclusions

μQFR showed significantly better agreement with FFR. The effect of abnormal cardiac structure, valvular regurgitation, and left ventricular diastolic function did not correlate with the diagnostic accuracy of μQFR. Coronary hemodynamics showed no difference in patients with abnormal cardiac structure, valvular regurgitation, and left ventricular diastolic function.

## Data Availability

The original contributions presented in the study are included in the article, further inquiries can be directed to the corresponding authors.
